# Determination of Pleiotropic Effect of Warfarin in *VKORC1* and *CYP2C9* Genotypes in Patients With Heart Valve Replacement

**DOI:** 10.3389/fcvm.2022.895169

**Published:** 2022-06-10

**Authors:** Huma Shafique, Naeem Mahmood Ashraf, Amir Rashid, Asifa Majeed, Tayyaba Afsar, Ann K. Daly, Ali Almajwal, Nawaf W. Alruwaili, Azmat Ullah Khan, Suhail Razak

**Affiliations:** ^1^Department of Biochemistry, Army Medical College, National University of Medical Sciences, Rawalpindi, Pakistan; ^2^Institute of Cellular Medicine, Newcastle University Medical School, Newcastle University, Newcastle upon Tyne, United Kingdom; ^3^Department of Biochemistry and Biotechnology, University of Gujrat, Gujrat, Pakistan; ^4^Department of Community Health Sciences, College of Applied Medical Sciences, King Saud University, Riyadh, Saudi Arabia

**Keywords:** *VKORC1*, *CYP2C9*, pleiotropy, IL-6, TNF-α, COX-2, TaqMan

## Abstract

Warfarin has been widely used as an oral anticoagulant agent. In past, efforts have been done to study the contribution of genetic variation on warfarin dose requirements. The possible therapeutic dose determination of warfarin is very challenging, i.e., extremely low dose leading to unusable antithrombotic therapy or high dose causes particularly bleeding complications. Our study aimed to investigate these observations in more detail, we determined the correlation of interleukin-6 (IL-6), cyclooxygenase-2 (COX-2), and tumor necrosis factor-α (TNF-α) among VKORC1 and CYP2C9 genetic variants in patients with heart valve replacement who were treated with a range of warfarin doses and compared with levels in healthy controls. A total of 107 human subjects were recruited with low < 5 mg, medium 5–10 mg/day, and high > 10 mg/day warfarin doses. The genetic study of VKORC1–1639G/A, C1173T, 3730G > A, CYP2C9*2, and CYP2C9*3 was performed using TaqMan genotyping and DNA sequencing. The gene expression of IL-6, TNF-α, and COX-2 mRNA was analyzed. IL-6, TNF-α, and COX-2 protein expressions were determined by ELISA and Western blot analysis to evaluate the pro- and anti-inflammatory effects of warfarin. A statistically significant difference was found among the haplotypes of VKORC1 rs9934438 (C1173T), rs9923231 (−1639G > A), rs7294 (3730G > A) and CYP2C9 *2 p. Arg144 Cys (rs28371674), CYP2C9 *3 p. Ile359Leu (rs1057910) genotypes with warfarin dose requirements (*p* = 0.001). The increased levels of COX-2, IL-6, and TNF-α proteins were observed when a high dose of warfarin (>10 mg/ml) was administered. However, a lower concentration (1.0 mg/ml) was observed with decreased warfarin dose (<5 mg/day). The present study reported that in addition to its anticoagulant action, the genetic variants of warfarin may have a pleiotropic effect by influencing IL-6 depending on the dosing regimen and inducing the expression of COX-2.

## Introduction

The polymorphisms in the noncoding regions of the *VKORC1* gene contributed to the variation in warfarin dose maintenance. The single nucleotide polymorphisms (SNPs) in noncoding regions are related to the warfarin dosages across the normal dose range (36 mg/week). The SNPs in the VKORC1 noncoding regions were found in various populations. The associated SNP of VKORC1, particularly −1639 > A and 1173 > T, were found to be located in a strong (LD) linkage disequilibrium region (1, 2). The type of polymorphism carrying the A allele has reduced VKORC1 mRNA manifested in around 90% of Asians and has marked variations in its percentage by ethnic group, and it appears to elucidate the low warfarin dose requirement. It is a universal finding that A allele carriers require a low initiation dose as compared to the G allele (3).

Warfarin is considered to affect the inflammatory pathway in addition to its anticoagulant action (4, 5). The dichotomic effect of warfarin means that at high concentrations, i.e., >200 mM of warfarin, high levels of interleukin-6 (IL-6) were observed in macrophage cells, therefore acting as pro-inflammatory, whereas at low warfarin concentrations, i.e., < 200 mM, there was inhibited tumor necrosis factor-α (TNF-α)-induced IL-6 release. This showed that these inflammatory activities might have significance *in vivo*. The best study effect of warfarin on inflammation is on IL-6 levels (6). In macrophages, warfarin treatment resulted in decreased IL-6 levels at low concentrations (7) and increased levels at higher concentrations (8). In addition, warfarin inhibited the stimulation of I κ-B phosphorylation by TNF-α, a pro-inflammatory cytokine. Moreover, variations in *VKORC1* were seen as related to dosing regimens of warfarin (9).

Some studies examined the possibility that the coagulation system can directly affect the inflammatory regulatory pathway (10). Warfarin may act by other molecular mechanisms apart from its effect on vitamin K metabolism (5). It affects the immune system involving IL-6 production, stimulated by TNF-α, and cyclooxygenase-2 (COX-2) may mediate this process because the levels of both IL-6 and prostaglandin E2 are elevated during inflammation (11). The dose of warfarin is an essential factor to upregulate and downregulate interacting cytokines, e.g., a low dose of warfarin (10–100 μM) inhibited the TNF-α-induced production of IL-6 in murine macrophages, whereas a high dose of warfarin (>200 μM) stimulated IL-6 release mediated by prostaglandins (9).

To investigate these observations in more detail, we determine the correlation of IL-6, COX-2, and TNF-α among VKORC1 and CYP2C9 genetic variants in heart valve replacement patients treated with a range of warfarin doses and compared with levels in healthy controls. In view of the previous finding, *in vitro* suggesting warfarin affects the response to TNF-α treatment (12). Warfarin affects the immune system involving IL-6 production, stimulated by TNF-α. COX-2 may be involved in mediating the process because the levels of both IL-6 and prostaglandin E2 are elevated during inflammation (13). Molecular dynamic (MD) simulation is an important tool to understand the effect of amino acid substitutions on protein structural and functional levels (14). Therefore, drug–protein docking analysis and MD simulations are also performed to get an insight into the effect of SNPs at expression and structural levels.

## Materials and Methods

### Study Design and Location

The study design was a prospective cohort. It was conducted at the Centre for Research in Experimental and Applied Medicine (CREAM), Army Medical College, National University of Sciences and Technology, Pakistan, in collaboration with the Institute of Cellular Medicine, Faculty of Medical Sciences, Newcastle University Medical School, United Kingdom.

### Sampling Technique

The non-probability convenience sampling technique was applied according to the warfarin dose–response relationship, i.e., patients who had a low dose (<5 mg/day), patients who had a medium dose (5–10 mg/day), and patients who had a high dose (>10 mg/day) of warfarin.

### Inclusion Criteria

Patients on long-term warfarin therapy for anticoagulation management were included.

### Exclusion Criteria

Patients on anticoagulants for short-term warfarin therapy for the treatment of other indications, such as pulmonary embolism, deep venous thrombosis, and atrial fibrillation, were excluded.

### Subjects

A total of 107 human subjects were included in the study, and out of which 43 (40.2%) were taking warfarin < 5 mg/day, 57 (53.3%) had 5–10 mg/day, and 7 (6.5%) were on > 15 mg/day. We obtained written informed consent from all patients and approval from the Institutional Ethical Review Board (IERB), Armed Forces Institute of Cardiology and, National Institute of heart diseases (AFIC-NIHD), Rawalpindi, Pakistan (15) as per the reference number AFIC-IERB-SOP-15. The patient’s medical history was obtained along with 30 healthy human subjects as controls on approved proforma by the clinical supervisor at AFIC-NIHD.

### Extraction of Genomic DNA From Human Blood

Genomic DNA extraction from blood was done by means of organic solvents (16). The DNA was concentrated (Vacuum Concentrator 5301, Germany) for about a few minutes and dissolved in 200 μ of Tris–EDTA (TE) buffer.

### Whole Genome Amplification

The whole genome amplification (WGA) method is used to amplify the entire genome (17). WGA samples were measured for TaqMan genotyping of VKORC1, CYP2C9 *2, and CYP2C9*3 SNPs.

### VKORC1 and CYP2C9 TaqMan Genotyping Assay

**FIGURE 1:**
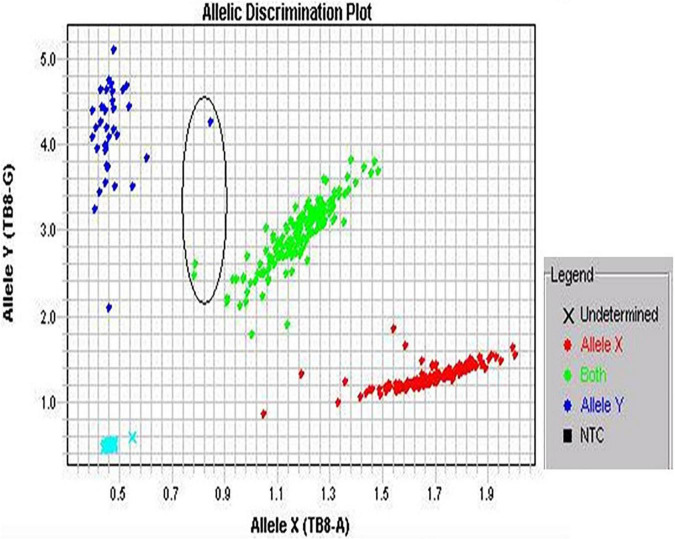
Screenshot of allelic discrimination plot. The three colored dots represent an individual genotype: blue and red dots show homozygotes, whereas green dots show heterozygotes. The encircled dots show not tightly clustered.

TaqMan^®^ genotyping analyses SNPs by means of 5′ nuclease analysis for amplifying specifically SNP alleles in pure genomic DNA samples (18). The assay consisted of basically two primers for amplification of DNA sequence and two minor groove binder (MGB) probes for identifying alleles. The 5′ ends of each MGB probe contained target-specific oligonucleotides with reporter dye VIC, FAM, and non-fluorescent quencher. The procedure was adopted as per the manufacturer’s instruction (Figure 1).

### RNA Extraction and cDNA Synthesis by Reverse Transcription

Total RNA was extracted from the blood within 6 h of collection by using PureLink RNA Mini Kit Ambion by (Life Technologies™; Thermo Fisher Scientific, United States) and was stored at −80°C. cDNA was synthesized using 1 μg of total RNA. For the synthesis reaction, 1 μl of random hexamers and deoxynucleoside triphosphate (dNTP, 10 mM) and 6 μl of diethylpyrocarbonate (DEPC) water were mixed and combined with 2 μl of RNA. The tubes were incubated at 65°C for 10 min and ice-chilled for 2 min. In total, 2 μl of 10X buffer, 0.25 μl of reverse transcriptase enzyme, 0.1 μl of RNase inhibitor, and 7.65 μl of DEPC water were added and incubated at 37°C for 50 min and at 70°C for 15 min.

### qPCR With TaqMan Gene Expression Assay

The expression analysis of IL-6, TNF-α, and COX-2 was performed by the quantitative real-time PCR TaqMan Expression Assay. The relative quantification (RQ) of the gene was done using glyceraldehyde-3-phosphate dehydrogenase (GAPDH). Samples were used in triplicates to minimize the error and for valid data interpretation. TaqMan expression assay was performed on Applied Biosystems StepOne™ Real-Time PCR System using 2X TaqManUniversal PCR Master Mix (Applied Biosystems). Thermocycling conditions were 10 min at 95°C, 40 cycles of denaturation at 95°C for 15 s, and annealing and extension for 1 min at 60°C. The relative fold change in the expression of the target gene was determined by 2^–ΔΔCt^ and analyzed using StepOne Software version 2.3. The primers used are shown in Table 1.

**TABLE 1:** List of primers.

Primers and SNPs	Sequence	Region	Reaction conditions (°C)	Product size
*VKORC1EX1F*	5′CAATCGCCGAGTCAGAGG 3′	Exon 1	56	338 bp
*VKORC1EX1R*	5′ TAATCATCTGGCATCCTGGC 3′			
*VKOREX2F*	5′ ACAGTCCTAACCTGGTTCCA 3′	Exon 2	59	280 bp
*VKOREX2R*	5′ TGAGCTGACCAAGGGGGATG 3′			
*VKOREX3F*	5′ TGAAGCCCACACCGGACCCT 3′	Exon 3	57	214 bp
*VKOREX3R*	5′ GCAAAGCAGATGAGGTCAGC 3′			
rs9923231F	3′GCCAGCAGGAGAGGGAAATA5	Promoter	58	290 bp
rs9923231R	3′AGTTTGGACTACAGGTGCCT5′			
rs7294F	3′GGCTTACGCACGTATTCC5′	3′	60	659 bp

### Quantification by ELISA

IL-6, COX-2, and TNF-α levels were determined in triplicate by the COX-2 ELISA Kit (Cat# 99-0062, Invitrogen) and the IL-6 and TNF-α ELSIA Kits (Cat# KAC1261 and Cat# KAC1751, respectively, Invitrogen, BioSource Europe S.A) according to the manufacturer’s instructions. The standard absorption was measured by linear regression, and the quantity of IL-6 was calculated by *R*^2^ = 0.99.

### HepG2 Cell Culture

The HepG2 cells were cultured in Dulbecco’s modified Eagle’s medium (DMEM) and added in 10% fetal bovine serum (FBS), 1 mM Na pyruvate, 2 mM L-glutamine, penicillin (100 units/ml), and streptomycin (100 mg/ml). The cells were seeded (105 cells/ml) and maintained in 75 cm^2^ flasks at 37°C in a 5.5% CO_2_ incubator. Warfarin stock was prepared in absolute ethanol and used in the culture medium to obtain the following final warfarin concentrations: 1.0 and 10 mg/ml. The culture medium was removed, and the monolayer was washed two times with phosphate-buffered saline (PBS, pre-warmed, pH 7.3), and cells were incubated in DMEM containing warfarin to get the final concentrations (1.0 and 10 mg/ml). The experimented cells were harvested by scraping the cells at 24 h. The scraped cells were transferred in conical tubes (15 ml), and 2 ml cold PBS was added, pelleted by centrifuging at 12,000 rpm for 10 min. The harvested cell pellets were frozen at −80°C.

### Western Blot Analysis

The IL-6, TNF-α, and COX-2 protein expression levels were also determined by Western blot analysis. The HepG2 cells (warfarin-exposed) were extracted using the Trizol method (Invitrogen) as per the manufacturer’s instructions. They were immersed in liquid nitrogen for instant freeze and kept in microcentrifuge tubes. The samples were kept at −80°C. Chilled lysis buffer of 300 μl was added in the tube, for a 5 mg piece of tissue, regulated by a homogenizer, and the blade was washed twice with 2X lysis buffer (200 μl). Continuous agitation was done at 4°C for 2 h and centrifuged (12,000 rpm 20 min at 4°C). Placed on ice, the supernatant was aspirated and shifted into a new tube, and the pellet was wasted. By using 10X protein gel buffer, 30 μg protein from cell homogenates were loaded on 15% sodium dodecyl sulfate (SDS) gel. The gel was run for 1–2 h at 100 V. Proteins were blotted on the nitrocellulose membrane, and 5% of milk mixed in 0.1% Tween-20 was used. The membrane washed with 0.1% Tween-20 and Rabbit polyclonal pre-albumin antibody was incubated overnight at 4°C.

After blocking with Rabbit Polyclonal Pre-albumin Antibody, the membrane was incubated from (ab16006) Abcam H, diluted in 1:3,000 dilution PBS with Tween-20 (PBS/T) at 4°C, and kept in rocker overnight. The membrane was rinsed three times with PBS/T for 10 min and incubated with Sigma-Aldrich H Goat Anti-Rabbit Ig HRP Conjugate, diluted in 1:7,500 dilutions in 5% non-fat dried milk PBS/T for 1 h. The membrane was rinsed again thrice after 10 min with the same wash buffer. By using SuperSignal West Pico Chemiluminescent Substrate, bands were developed in CL-XPosure Film. For control, we used Anti-GAPDH antibody produced in rabbit [Sigma-Aldrich H (G9545)].^1^

### *In silico* Analysis of Variants

*In silico* analysis of the variants of both genes was performed to get further insight. The three-dimensional structure of CYP2C9 (1OG2) was downloaded from the RCSB Protein Data Bank. The structure was cleaned by removing the water molecules for further bioinformatics analysis. The three-dimensional structure of warfarin was downloaded from PubChem database, and its energy minimization was carried out in Chem3D Pro (version 12.0). The mutants of CYP2C9 were generated using PyMOL. The protein–ligand docking analysis was carried out using CB-Dock online server. The CB-Docked uses Vina for protein–ligand docking (14). The best docking pose was selected based on the Vina score (19). The MD simulations were performed to check the binding of warfarin with the wild-type and mutant CYP2C9 dynamically. GROMACS 5.0.5 version was used to perform MD simulations (20). A total of 100 ps of the equilibration phase was followed by 10 ns of the production phase. Finally, the wild-type and mutant trajectories were used to calculate the root mean square deviation (RMSD), root mean square fluctuations (RMSF), the number of hydrogen bonds (HBs), and drug–protein interaction energy. The functional impact of non-coding SNPs in VKORC1 was analyzed using SNPinfo (FuncPred) using the Asian population’s web servers (21).

### Statistical Analysis

Descriptive statistics was calculated for high warfarin doses (>10 mg/day). The mean ± SD of variables was calculated. Pearson’s coefficient was calculated to study the dose–response relationship of warfarin with IL-6, COX-2, and TNF-α expression. The mean ± SD was calculated for IL-6, TNF-α, and COX-2 levels. SPSS version 22.0 was used to find statistical significance at *p* ≤ 0.05.

## Results

### Genotypes Frequencies Using Hardy–Weinberg Equilibrium in a Studied Population

Out of the total, 107 Pakistani patients with heart valve problems (congenital heart valve disease, mitral, aortic, pulmonary, and tricuspid valve stenosis) were treated by surgery (mitral, aortic, tricuspid valve replacements, and repaired) and put on anticoagulation. The genotype frequencies of *VKORC1* 1639G > A (rs9923231), C1173T (rs9934438), 3730G > A (rs7294) and *CYP2C9* *2 p. Arg144Cys (rs28371674), *CYP2C9* *3 p. Ile359Leu (rs1057910) SNPs were calculated using Hardy–Weinberg equilibrium (HWE) in Pakistani subjects. These SNPs were also observed in other subjects. The selected population followed the HWE.

### *VKORC1* SNPs: 1639G>A (rs9923231), C1173T (rs9934438), and 3730G>A (rs7294)

#### SNP1: 1639G>A (rs9923231)

**FIGURE 2:**
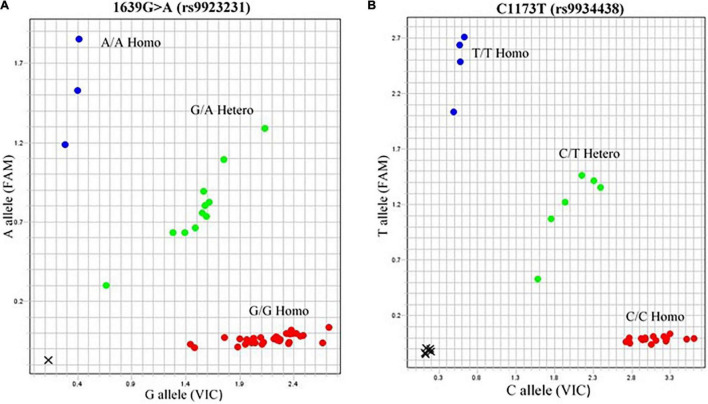
Allelogram showing *VKORC1* SNPs. 1639G > A (rs9923231) and C1173T (rs9934438) represented as heterozygous GA and CT phenotypes are in green dots obtained by TaqMan SNP genotyping assay. The genotype of samples of rs9923231 was further confirmed by direct sequencing.

Genotype frequencies of *VKORC1* (GG, GA, and AA) were observed in Pakistani patients receiving warfarin. Maximum 1639G>A polymorphisms were noted in 48% Punjabis, 17% Pathans, and 15% Sindhis. The percentage of genotype (GG, GA, and AA) frequency in Punjabis noted was 21% GG, 19% GA, and 8% AA. In the case of Pathans, the GG, GA, and AA genotype frequency was 7, 4, and 6%, respectively. The frequency in Sindhi subjects who reported GG genotype was 11%, 5% GA, and 3% AA (Table 4). The frequency did not deviate from HWE (Figure 2A).

#### SNP2: C1173T (rs9934438)

The genotypic frequencies were observed in the distribution of three genotypes (CC, CT, and TT). The genotype frequency found in Punjabis for CC, CT, and TT was 13, 11, and 7%, respectively, whereas Pathans had 3% CC, 6% CT, and 3% TT genotype frequency. The Sindhi subjects had 14% CC, 9% CT, and 19% TT genotype frequency (Figure 2B).

#### SNP 3: 3730G>A (rs7294)

**FIGURE 3:**
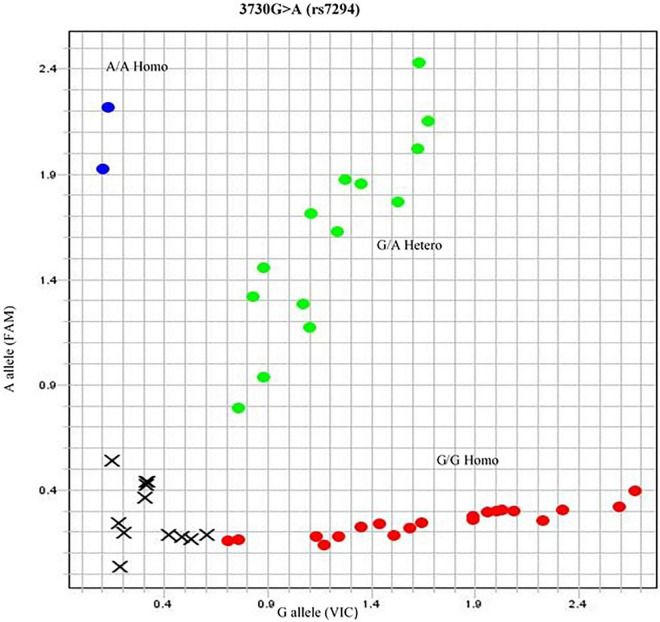
Allelogram showing 3730G > A (rs7294) SNP. The SNP represented in red dotted area as homozygous GG phenotype obtained by TaqMan SNP genotyping assay. The genotype of samples of rs7294 was further confirmed by direct sequencing.

The association of genotype frequency (GG, GA, AA) was within the HWE among the ethnicity of the population (*p* = 0.373). In Punjabis, the genotype frequency reported for GG and AA was 3 and 1%, respectively. In the case of Pathans, 1% GG and 1% AA genotype frequency were recorded. The Sindhi subjects had 3% GG and 2% AA genotype frequency, respectively. In the case of British population, the GA genotype was 3% (Figure 3).

### *CYP2C9* SNPS

#### SNP 1: CYP2C9*2p. Arg144Cys (rs28371674)

**FIGURE 4:**
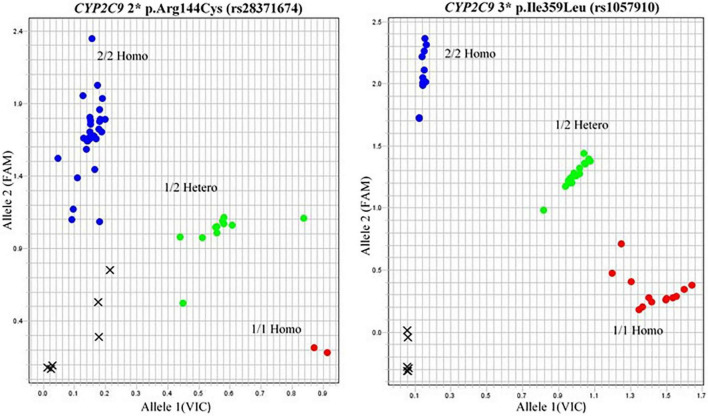
Allelogram showing *CYP2C9* 2* and 3* SNP genotyping in the blue dotted area.

In the case of *CYP2C9* polymorphism, the wild type *1 was compared with *2 variants from subjects belonging to different regions. The frequency was 28% in the case of Punjabi patients receiving warfarin therapy as compared to the *1 wild type (48%), whereas 9% of Pathans and 13% of Sindhis had *2 *CYP2C9* variant phenotype, respectively. In the case of 6% Indians, 9% Thai, and 5% British had *2 *CYP2C9* genotype (Figure 4).

#### SNP 2: *CYP2C9* *3p. Ile359Leu (rs1057910)

*CYP2C9* *3 phenotype was observed in 6% of the Punjabi population, 1% of the Pathans, and 9% of the Sindhis. No *3 *CYP2C9* phenotype was recorded in the Thai and British groups.

The Allelogram showed three distributed tightly clustered colored dots for comparison of homozygous and heterozygous alleles with the wild type (Figure 4). The red dots depicted the samples carrying wild-type alleles. Green dots indicated the heterozygous GA/CT alleles in the case of *VKORC1* genotypes and *2 alleles of *CYP2C9*. Blue dots showed the AA/TT homozygous of *VKORC1* and *3 alleles in the case of *CYP2C9*. The cross closed to a 0.0 value of the plot showed the undetermined data (Figure 4).

### Association of Cytokine Levels and VKORC1 and CYP2C9 Genotypes With Warfarin Dosage Groups

#### Low-Dose (<5 mg/Day) Warfarin Group

The SNPs reported in VKORC1 have been correlated with warfarin dose requirement. In the case of SNP 1639G>A, significant differences were observed in the distribution of the three genotypes (GG, GA, and AA). The VKORC1 (1639G>A) AA homozygous with CYP2C9 *1/*3 variant alleles were associated with a low warfarin dose (1.25 ± 0.34, *p* = 0.032). A significantly lower mean warfarin dose (2.5 ± 0, *p* = 0.001) was recorded in patients with VKORC1 (1639G>A) GA homozygous with CYP2C9 *2/*3 variant alleles as compared to the wild type.

The variant allele Ct (cycle threshold) of VKORC1 (1173C>T) with CYP2C9 *1/*2 and *2/*3 showed a lower mean warfarin dose (2.3 ± 0.23, *p* = 0.04 and 1.5 ± 0, *p* = 0.02, respectively). A mean warfarin dose of 1.27 ± 0.38 with *p* = 0.015 was recorded in the TT variant with *3*3 CYP2C9 genotype. The INR was recorded to be >4 in these subjects.

The VKORC1 (3730G>A) AA variant with *1*3 CYP2C9 genotype. The genotype showed a significantly low warfarin dose (1.55 ± 0.64, *p* = 0.03).

The Ct values of cytokines were reported by qPCR where the primary curve was crossing the threshold. The mean Ct value was calculated for all samples of the dosage groups in Pakistani subjects. In the case of the low-dose group (<5 mg/day), the VKORC1 (1639G>A) AA homozygous with CYP2C9 *1/*3 variant allele showed a mean Ct value of *IL-6* (27.03 ± 0.06), i.e., 0.45-fold lower expression interpreted 2-fold downregulated. The mean Ct for *TNF-α* was 27.28 ± 0.09 with a 0.37-fold decrease, i.e., 2.7-fold downregulation was recorded. *COX-2* had a Ct value of 26.25 ± 0.07 with 0.27-fold difference interpreted to be 3.7-fold downregulated as compared to the control (Table 2).

**TABLE 2:** Comparison of fold change expression of target genes in low and high warfarin dose groups calculated by ΔΔCT method in Pakistani subjects.

Warfarin dose gps	Expression levels	Target mean C_t_	(GAPDH) mean C_t_	Target-control Δ Ct	ΔΔ Ct	Fold difference
Low dose gp <5 mg/day	Control	30.49 ± 0.15	23.63 ± 0.09	6.86 ± 0.19	0.00 ± 0.19	0.9–1.1
	IL-6	27.03 ± 0.06	22.66 ± 0.08	4.37 ± 0.10	−2.4 ± 0.10	5.3–6.0
	TNF-α	27.28 ± 0.09	23.5 ± 0.07	3.78 ± 0.20	−2.8 ± 0.20	4.3–5.0
	COX-2	26.25 ± 0.07	24.60 ± 0.07	1.65 ± 0.10	−5.11 ± 0.10	34.5–39.7
High dose gp >10 mg/day	Control	26.47 ± 6.21	18.62 ± 0.52	7.68 ± 6.2	5.69 ± 6.2	0.00028–1.5
	IL-6	19.78 ± 5.25	18.62 ± 0.52	0.07 ± 3.7	−1.49 ± 3.7	0.027–0.2
	TNF-α	18.69 ± 3.76	18.22 ± 0.72	1.56 ± 5.2	0.0 ± 5.2	0.0272–36.75
	COX-2	18.28 ± 2.98	17.11 ± 0.31	1.17 ± 4.8	0.1 ± 4.9	0.038–32.9

#### Medium-Dose (5–10 mg/Day) Warfarin Group

Patients recorded under this group had a mean warfarin dose of 5.65 ± 0.74, *p* = 0.01 of *VKORC1* (1639G>A) GG homozygous with CYP2C9 *1/*2 variant alleles The CC homozygous of VKORC1 (1173C>T) with CYP2C9 *1/*1 showed a mean dose of 5.84 ± 1.23, *p* = 0.03.

The GG homozygous of VKORC1 (3730G>A) with CYP2C9 *1/*2 had a mean value of 7 ± 0, *p* = 0.012 as reported. The international normalized ratio (INR) of these patients with medium warfarin doses (5–10 mg/day) lies within the therapeutic range (2.0–3.5).

#### High-Dose (>10 mg/Day) Warfarin Group

The GG/GA homozygous of VKORC1 (3730G>A) is a variant associated with high warfarin dosage. The GG genotype of VKORC1 (1639G>A) with CYP2C9 *1/*1 was recorded with a mean warfarin dose of 8.52 ± 2.4 (*p* = 0.001). In case of other subjects, the mean dose required for GA genotype with CYP2C9 *1/*1 was 13.6 ± 3.05, *p* = 0.01. For high warfarin dose group, the average Ct value of VKORC1 GA genotype with CYP2C9 *1/*1 showed *IL-6* as 19.78 ± 5.25 with a 1.78-fold increase. TNF-α had 18.69 ± 3.76 Ct value with 1.67-fold expression, and COX-2 had 18.28 ± 2.98 with 1.7-fold expression (Table 2).

### Correlation Between Cytokine Levels and Warfarin Dosage

The significant mRNA results of cytokine levels were evaluated with ELISA using serum samples of human subjects. A significant positive correlation was found between IL-6 and warfarin (correlation coefficient: 0.68, *p* < 0.05), COX-2 and warfarin (correlation coefficient: 0.69, *p* < 0.05), and TNF-α and warfarin dose (correlation coefficient: 0.57, *p* < 0.05). The consistently increased levels of IL-6, TNF-α, and COX-2 were observed along with increased warfarin doses (2.5, 5, 7.5, 10, 12.5, and 15) mg/day (Figure 2C). The results showed the decreased levels of IL-6, TNF-α, and COX-2 at a low dose (2.5–5 mg/day) and significantly increased IL-6, TNF-α, and COX-2 levels at a high dose (12.5–15 mg/day) (Table 3).

**TABLE 3:** Relationship between IL-6, COX-2, and TNF-α.

Characteristic s	IL-6 conc of a patient	COX-2 conc of a patient	TNF-α conc of a patient
IL-6 conc	1	0.993**	0.993**
COX-2	0.993**	1	0.997**
TNF-α	0.993**	0.997**	1

*A strong positive correlation was noted between IL-6 and COX-2 (correlation coefficient: 0.993, p < 0.05) levels. A positive significant correlation was observed between TNF-α and COX-2 (correlation coefficient: 0.997, p < 0.05) levels.*

**Indicate p < 0.01.

**TABLE 4:** The increased levels of IL-6 observed in HeLa cells on varying warfarin concentration.

Warfarin concentration (μM)	IL-6 production (pg/ml) during time of treatment (h)		
	4 h	8 h	16 h
Control without IFNγ	17.8 ± 1.28	18.3 ± 0.84	20.3 ± 0.62
Control with IFNγ	24.6 ± 1.69	36.6 ± 1.69	46.6 ± 2.05
20	19.16 ± 1.64	24.6 ± 1.24	26.7 ± 0.23
50	32.3 ± 1.02	38.5 ± 0.7	43.8 ± 0.62
100	45.1 ± 1.31	48.16 ± 3.11	52.8 ± 1.02
150	59.1 ± 4.64	63.8 ± 2.45	69.8 ± 4.6
200	74.9 ± 3.26	80.6 ± 4.58	83.56 ± 2.04
250	94.36 ± 6.62	107.3 ± 3.68	113.5 ± 5.57

### Effect of Warfarin on *IL-6*, *TNF-α*, and *COX-2* Gene Expression

**FIGURE 5:**
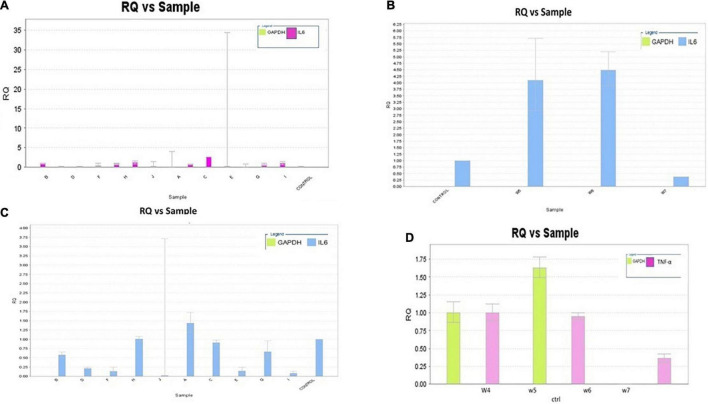
**(A)** IL-6 mRNA expression on low warfarin dose. Samples are plotted on the *x*-axis and RQ on the *y*-axis. Each bar shows the set of sample values of a detector. The detector for the *IL-6* gene is shown in purple. The error bars show the RQ min/max expression levels that measured the standard error of the mean expression level (RQ value). **(B)** IL-6 mRNA expression on medium warfarin dose. The detector for *IL-6* gene is shown in blue. The error bars show the RQ min/max expression levels that measure the standard error of the mean expression level (RQ value). **(C)** IL-6 mRNA expression on high warfarin dose. Samples are plotted on the x-axis and RQ on the *y*-axis. Each bar shows the set of sample values of a detector. The detector for the IL-6 gene is shown in blue. The error bars show the RQ min/max expression levels that measured the standard error of the mean expression level (RQ value). **(D)** TNF-α mRNA expression on high warfarin dose. The detector for the TNF-α gene is shown in purple. The error bars show the RQ min/max expression levels that measured the standard error of the mean expression level (RQ value).

**FIGURE 6:**
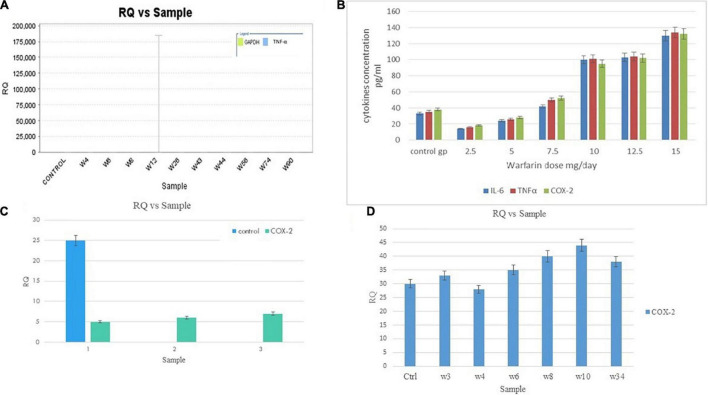
**(A)** TNF-α mRNA expression on low warfarin dose. Samples are plotted on the *x*-axis and RQ on the *y*-axis. Each bar shows the set of sample values of a detector. The detector for the *TNF-α* gene is shown in blue. The error bars show the RQ min/max expression levels that measured the standard error of the mean expression level (RQ value). **(B)** COX-2 mRNA expression on low warfarin dose. The detector for *COX-2* gene is shown in green. The error bars show the RQ min/max expression levels that measured the standard error of the mean expression level (RQ value). **(C)** Association of IL-6, COX-2, and TNF-α serum levels with warfarin dose: Serum IL-6, COX-2, and TNF-α levels were detected by ELISA with different warfarin doses (2.5, 5, 7.5, 10, 12.5, and 15) mg/day and compared with Control. **(D)** COX-2 mRNA Expression on high warfarin dose. Samples are plotted on the *x*-axis and RQ on the *y*-axis. Each bar shows the set of sample values of a detector. The detector for the *COX*-2 gene is shown in blue. The error bars show the RQ min/max expression levels that measured the standard error of the mean expression level (RQ value).

The RQ vs. samples of *IL-6*, *TNF-α*, and *COX-2* gene expression was recorded and compared low and high warfarin doses with endogenous control. The RQ values were plotted as (Log of RQ); therefore, RQ was adjusted to 1. The IL-6, TNF-α, and COX-2 expression levels were normalized to that of the reference control. Because the RQ was plotted on a log10 scale, it was displayed as 0 (log10 of 1 = 0) (Figure 5). The error bars shown in the graphs measured the RQ maximum and RQ minimum expression levels representing the standard error of the mean expression level based on the confidence level in RQ (Min/Max) calculations. For example, for sample E, the IL-6 detector for low warfarin dose was detected with a high Ct value of 36.7 ± 5.194, with RQ value 0.035 and RQ min/max value recorded to be 0/184.031 confidence interval (Figures 5A–C). The sample w6 for the IL-6 detector showed a strong positive Ct value of 24 ± 0.062 with RQ value 1.433, and RQ min/max value was recorded to be 1.192/1.723 confidence interval (Figure 5C). In the case of TNF-α, the sample w6 for high warfarin dose showed a positive Ct value of 23.1 ± 0.025, RQ = 0.91, and RQ min/max = 0.845/0.98 (Figure 5D), and in sample w12 for low warfarin dose, a very high Ct value was recorded that is 40.33 ± 1.709, indicating a very low expression level with RQ = 0.023 and RQ min/max = 0/3.7 (Figure 6A). Similarly, for COX-2 expression level, sample w1 was noted with a high Ct value of 33.27 ± 0.173 with RQ = 0.082 and RQ min/max = 0.049/0.137 for low warfarin dose (Figure 6B). For sample w10, positive Ct value of 23.9 ± 0.039, RQ = 1.78 (Figure 6C), and RQ min/max = 1.58/1.9 for high warfarin dose requirement was observed (Figure 6D).

### Identification of Protein Levels With Warfarin Dose

**FIGURE 7:**
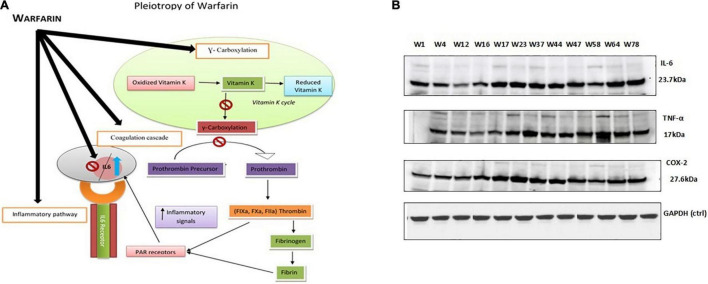
**(A)** Proposed model of warfarin’s pleiotropic effect: Illustrates that warfarin either in a low dose or a high dose inhibits the effect of the vitamin K cycle and thus gamma carboxylation in a controlled manner. Therefore, prolonging the coagulation pathway. The process of gamma carboxylation and inflammatory pathway goes side by side; thus, warfarin if affecting the coagulation pathway also influences the inflammatory pathway by directly inhibiting or stimulating the interacting cytokine levels. **(B)** Western blot protein expression analysis: IL-6 (23 kDa), TNF-α (17 kDa), and COX-2 (27 kDa) protein expression levels with a higher dose (>10 mg/day) of warfarin drug along with endogenous control GAPDH (37 kDa).

The same results were obtained by performing ELISA and Western blot through cell culture. The findings revealed that warfarin suppressed the IL-6 at a lower concentration (1 mg/ml) and acted as pro-inflammatory greater than 10 mg/ml (Table 2 and Figure 7).

### Effect of Warfarin on HepG2 Cell

The genetic variants selected for comparison were *VKORC1* GA genotype with *CYP2C9* *1/*1 associated with high dose. The bands on the gel for the selected higher dose genotypes (w1, w4, w12, w16, w17, w23, w37, w44, w47, w58, w64, and w78) clearly demonstrated the effect of warfarin drug on the respective protein expression (Figure 3B). As compared with control at 0 h, a fold difference of 2.8 in IL-6, 2.6 in TNF-α, and 2.3 in COX-2 protein expression was observed in cells exposed to high warfarin concentration at 24 h. The result demonstrated the increased expression of IL-6, COX-2, and TNF-α protein expression level in a higher concentration of warfarin (10 mg/ml) compared with the expression level of GAPDH control (Table 4).

### Bioinformatic Analysis of Variants

**FIGURE 8:**
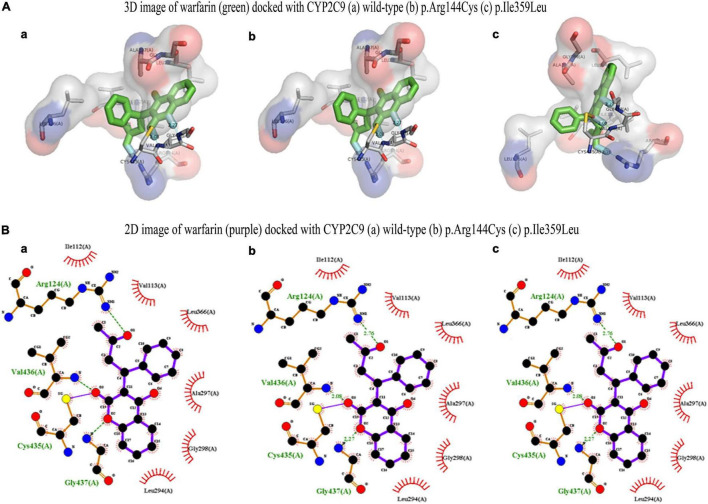
**(A)** 3D image of warfarin (green) docked with CYP2C9 (a) wild type, (b) *p.Arg144Cys*, and (c) p.Ile359Leu. **(B)** 2D image of warfarin (purple) docked with CYP2C9 (a) wild type, (b) *p.Arg144Cys*, and (c) p.Ile359Leu.

CYP2C9 plays a critical role to metabolize warfarin. The polymorphism associated with the gene is reported to confer a high risk of over warfarin-induced hemorrhagic complications (22). Docking and MD analysis of CYP2C9 wild-type and variants were performed with warfarin to get further insight and to support the wet-lab data. Docking of the wild type and mutants with the drug was not significantly different. The wild type and mutants showed the same pose and binding energy (−8.6 kJ/mol) (Figures 8A,B; 22). This change may affect the expression of the *VKORC1* gene. The change in expression level may bring a variable dose–response relationship.

**FIGURE 9:**
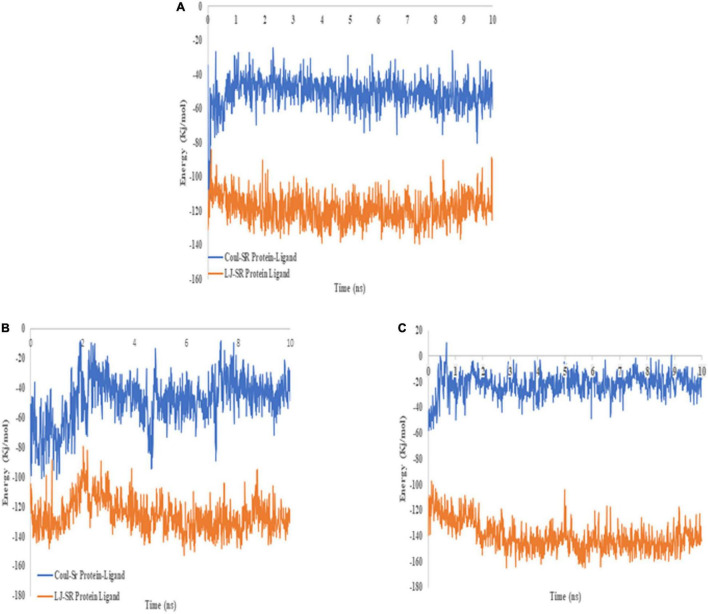
2D image of warfarin (blue) docked with CYP2C9 **(A)** wild type, **(B)** *p.Arg144Cys*, and **(C)** p.Ile359Leu. Hydrogen bonds are shown in orange, whereas eye-lashes are indicating hydrophobic interactions.

The dynamic analysis of docked complex revealed the distal effect of amino acid substitution. Binding energies calculated during MD trajectory analysis revealed that wild-type CYP2C9 (Coul-SR −50.79 kJ/mol) have better binding affinities for warfarin compared to the mutants (p. Arg144Cys Coul-SR −48.59 kJ/mol, p. Ile359Leu −21.88 kJ/mol) (Figures 9A–C).

**FIGURE 10:**
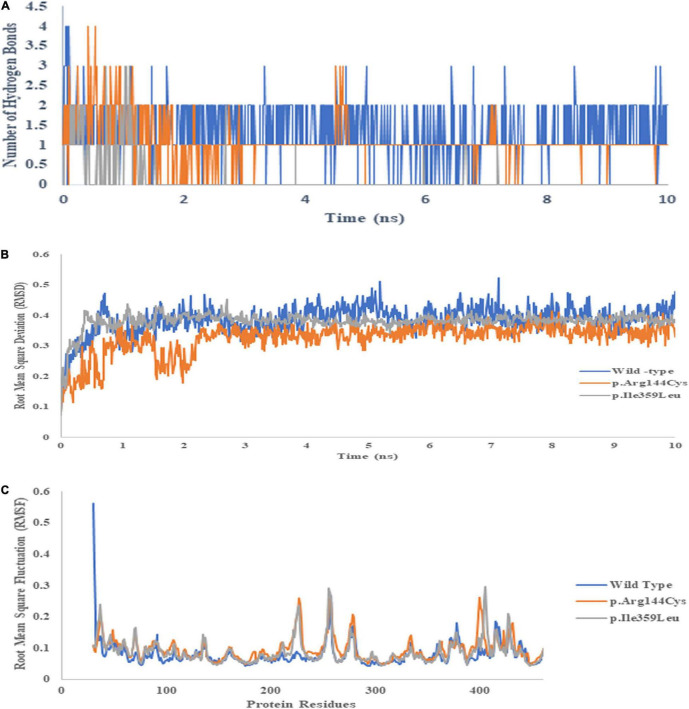
Binding energies of warfarin with the wild type **(A)**, *p.Arg144Cys* **(B)**, and p.Ile359Leu **(C)** derived from 10 ns MD trajectory analysis. The blue line represents Coul–SR interactions, whereas the orange line depicts LJ–SR interactions.

Similarly, hydrogen bond formation during the 10 ns simulation also showed the more hydrogen bonds of the wild-type protein with the drug compared to both mutants during the MD simulations (Figure 10A). The data suggest that better binding of warfarin with CYP2C9 may lead to fast metabolism and elimination of the drug from the body. p. Arg144Cys and p. Ile359Leu variants may lead to a delay in the elimination of the drug. Thus, they may significantly increase the risk of over-anticoagulation and hemorrhagic complications. There is no significant difference in RMSD (Figure 10B) and RMSF (Figure 10C) of the wild type and the mutants during 10 ns simulations. The functional analysis of non-coding SNPs of *VKORC1* genes (rs9934438, rs9923231, and rs7294) through the SNP info FuncPred tool revealed that these non-coding SNPs may disturb the transcription factor binding sites (TFBS) (22).

## Discussion

Warfarin drug is known to be the mainstay of an anticoagulation treatment worldwide. Clinical trials have established the efficiency of warfarin for treating thromboembolic illnesses. In addition to its effect as oral anticoagulation, warfarin may employ other alternative molecular actions that are said to be the pleiotropic effect of warfarin. Previous work done in 1979 by Eichbaum and his colleagues on experimental rodents demonstrated the inflammatory effect of warfarin but left with some controversies (23). Later in 2002, Kater and his colleagues tried to seek out, that warfarin inhibits inflammatory signal transduction at different concentrations (5). Therefore, we have focused on human subjects considering both warfarin-sensitive and warfarin-resistant patients and investigated the effect of warfarin drugs on IL-6 levels with TNF-α and COX-2 in patients with heart valve replacement by comparing them with controls.

The frequencies of three *VKORC1* SNPs (−1639G/A, 1173C/T, 3730 G/A) investigated in Pakistani subjects found that the genotypic differences led to the variances in warfarin maintenance dose. In the case of *VKORC1* SNPs detected in this study, patients with the A or T haplotype made a smaller amount of *VKORC1* than patients with the G or C haplotype, low doses were required to achieve an anticoagulant effect. The homozygous AA or TT variant alleles had less VKORC1 enzyme to function and low availability of vitamin K hydroquinone in cells, therefore causing the reduction in effective blood clotting. In patients of heterozygous GA/CT genotype, the VKORC1 enzyme activity is transitional and required high maintenance dose.

It is very important to study the genotypes of the *CYP2C9* gene to understand the warfarin metabolism (24). The pleiotropic effect of warfarin helped clinicians to adjust the appropriate dosage of NSAIDs or anti-inflammatory drugs.

In order to increase the safety and efficiency of warfarin in patients, the initial dose should be virtually decided based on SNP genotyping of *CYP2C9* and *VKORC1* genes (25). The genotype should be measured before initiating oral-anticoagulant therapies. The study proposed that the patient’s maintenance dose of oral anticoagulant is dependent on IL-6, TNF-α, and COX-2 levels along with VKORC1 and CYP2C9 genotypes. Therefore, to improve health care, not only the differences between the genotypes of individuals should be considered but also the differences in genotypes among different populations should be considered.

Modification in the effects of warfarin intake on TNF-α and IL-6 was observed, depending on peripheral blood leukocytes and on the cytokines studied (26). A controversy about COX-2, in several studies, shows that its action toward cellular IL-6 production is stimulatory or inhibitory, but this misperception is restricted to cell types. In this study, the COX-2 levels showed stimulatory action along with IL-6 production. Therefore, this study examined the IL-6 levels associated with different dosing schedules of warfarin and TNF-α and COX-2 mRNA and protein expression levels. In relation to warfarin to the immune system, the initiation of the blood coagulation cascade can activate an intracellular inflammation-signaling pathway depending on the dosing regimen of warfarin.

The significant positive correlation found between IL-6 and COX-2 levels showed that COX-2 enzyme functions parallel with the TNF-α stimulated IL-6 (27). The data were further supported by confirming a positive correlation between COX-2 and warfarin, IL-6 and warfarin, and TNF-α and warfarin (11). From our results, warfarin at low doses downregulated the expression of IL-6 and COX-2, and at higher doses, warfarin upregulated the expression levels of IL-6, TNF-α, and COX-2.

Warfarin’s inflammatory action is independent of its effect as a vitamin K antagonist. The exact regulatory mechanism of this mutual relationship needs to be further studied. However, we already published results of warfarin patients’ demographic and clinical variables interacting with IL-6 (28).

The results of protein expression levels parallel the mRNA expression of *IL-6*, *TNF-α*, and *COX-2* with a greater dose of warfarin (>15 mg/day) compared with the endogenous control GAPDH.

TNF-α and IL-6 as inflammatory factors have double effects, which may have immunomodulatory and anti-inflammatory (29). The active interaction may occur between the two processes of warfarin as anti-coagulation and inflammation (10). The significant proof of correlation supported a role in its inflammation. This proposes that an adjustable dose of warfarin may be required for its anti-inflammatory and pro-inflammatory efficacy. Warfarin may induce the expression of COX-2, thus causing increased prostaglandins during inflammation. The present study is the first of its kind that reported an association between genetic variants of warfarin with its dose requirement and certain interacting cytokines and COX-2 levels.

Molecular docking and MD simulations are important tools to understand drug–protein interactions. These tools help to understand the important pharmacodynamic properties (19). Sometimes, amino acid substitutions present at quite a large distance from the drug binding site may influence the drug–protein interactions. This study reveals a similar phenomenon. p. Arg144Cys and p. Ile359Leu amino acid substitution in the CYP2C9 results in weak interactions of the drug. The hydrogen bond plays an important role in the drug–protein interaction. MD data show a smaller number of hydrogen bonds of the drug in the variants as compared to the wild type during 10 ns simulations. It may lead to lower metabolism of the drug and delay in the drug plasma clearance.

Due to the pleiotropic properties of warfarin, this study may help the clinicians to adjust the appropriate doses of warfarin along with other drugs as anti-inflammatories (depending on its low concentration as anti-inflammatory and high concentration as pro-inflammatory) by comparing the interacting cytokine levels before and after surgery in heart valve replacement cases.

The study has a few limitations. INR values for the time of blood collection should be measured exactly along with interacting cytokines at the same time, which may give better results of warfarin action than dose. This needs to be evaluated in further studies, we were unable to justify it because of the lack of information from our patients.

## Conclusion

The study showed the altered effect of warfarin, other than its anti-coagulant action is correlated with inflammatory cytokines in patients on long-term dose therapy. This may be independent of the fact that the inflammatory action of warfarin was unknown before, or it may have altered long-term use by such patients. Therefore, the effect may help the clinicians to plan for a different strategy of treatment in warfarin therapy patients and hence save expenses of additional operative procedures and improve clinical outcomes.

## Data Availability Statement

The datasets presented in this study are included in the article/Supplementary Material.

## Ethics Statement

The studies involving human participants were reviewed and approved by informed written consent was taken from all patients. Approval from the Ethical Committee, Armed Forces Institute of Cardiology (AFIC-IERB-SOP-15), Rawalpindi was obtained. This study makes use of humans, and the experimental protocol for the use of humans was approved. The patients/participants provided their written informed consent to participate in this study.

## Author Contributions

HS, AM, NMA, AD, and AR together designed the research project. HS and AA performed the experiments. TA, AK, NWA, and SR analyzed the data along with AM. SR, TA, NWA, and AA wrote the manuscript. AM and AD were co-supervisors. AA provided technical assistance and improved the intellectual context of the research article. AR, AD, and SR supervised the project and reviewed the manuscript. All authors read and approved the final manuscript.

## Conflict of Interest

The authors declare that the research was conducted in the absence of any commercial or financial relationships that could be construed as a potential conflict of interest.

## Publisher’s Note

All claims expressed in this article are solely those of the authors and do not necessarily represent those of their affiliated organizations, or those of the publisher, the editors and the reviewers. Any product that may be evaluated in this article, or claim that may be made by its manufacturer, is not guaranteed or endorsed by the publisher.

## Footnotes

1
https://www.nacalai.co.jp/global/download/pdf/Western_Blot_Protocols.pdf
