# Supplementary material for: Determination of Pleiotropic Effect of Warfarin in VKORC1 and CYP2C9 Genotypes in Patients With Heart Valve Replacement
Source: Front Cardiovasc Med. 2022 Jun 10;9:895169. doi: 10.3389/fcvm.2022.895169 (PMC9226342; doi:10.3389/fcvm.2022.895169)
Supplement: Supplementary file 1 [file Data_Sheet_1.PDF]

Supplementary file S1

Investigating the pro- and anti-inflammatory effect of warfarin in *VKORC1* and *CYP2C9* genetic variants in heart valve replacement patients

Huma Shafique[1,4], Naeem Mahmood Ashraf[2], Amir Rashid[1], Asifa Majeed[1], Tayyaba Afsar[4], Anna Daly[5,] Ali Almajwal[4,] Nawaf W. Alruwaili[4], , Azmat Ullah Khan[2], Suhail Razak[,3, 4*]

IL-6

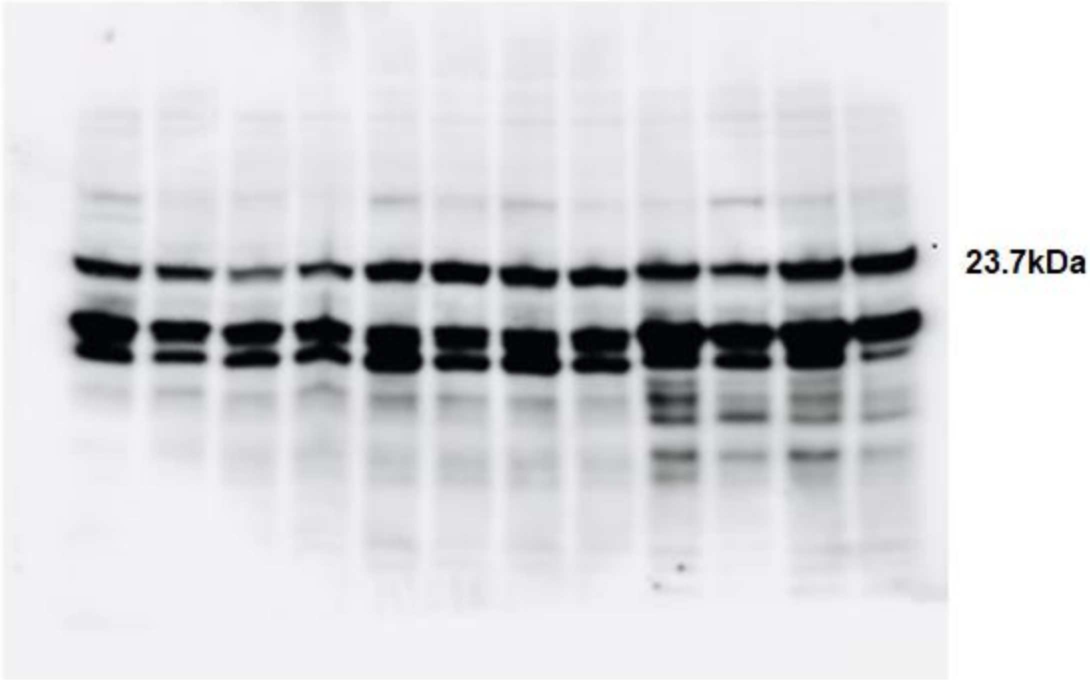

TNF-α

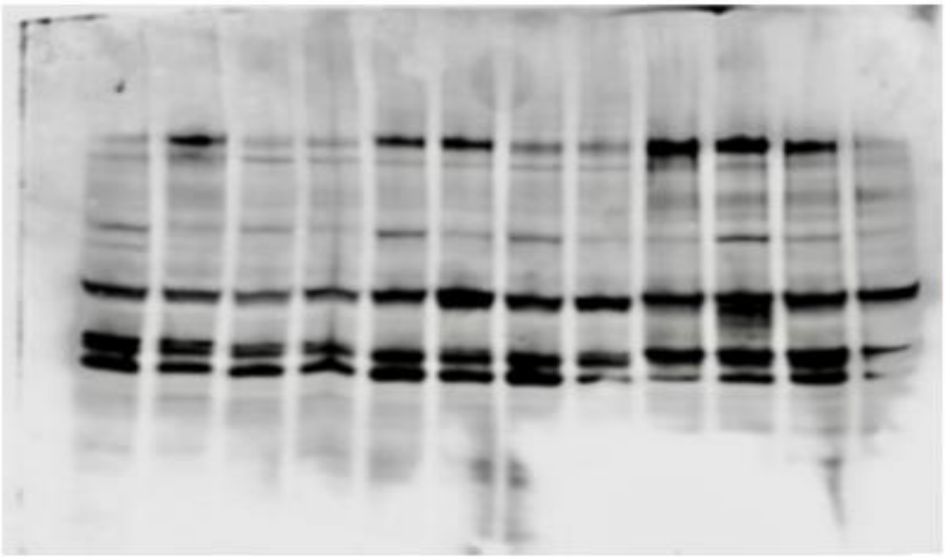

17kDa

cox-2

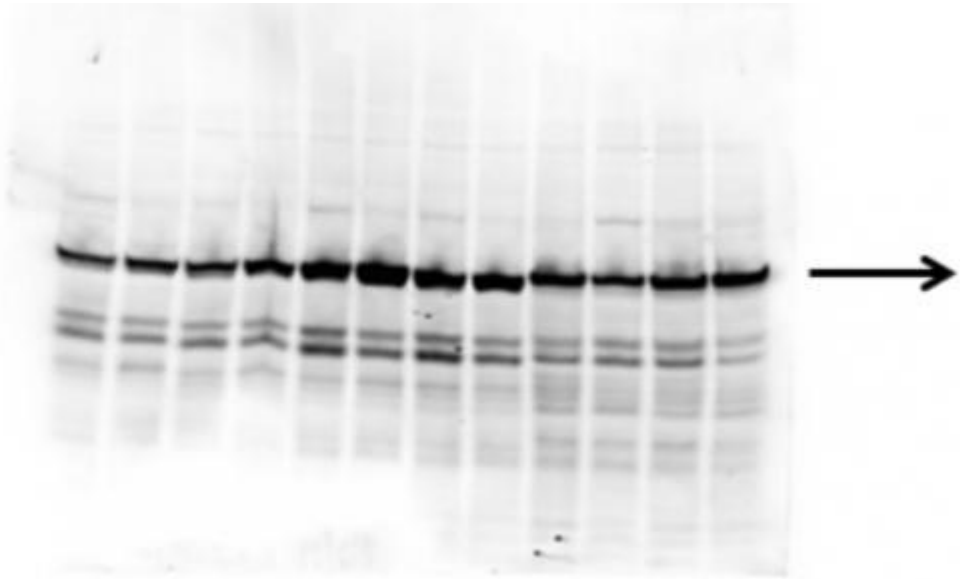

27.6 kDa

GADPH

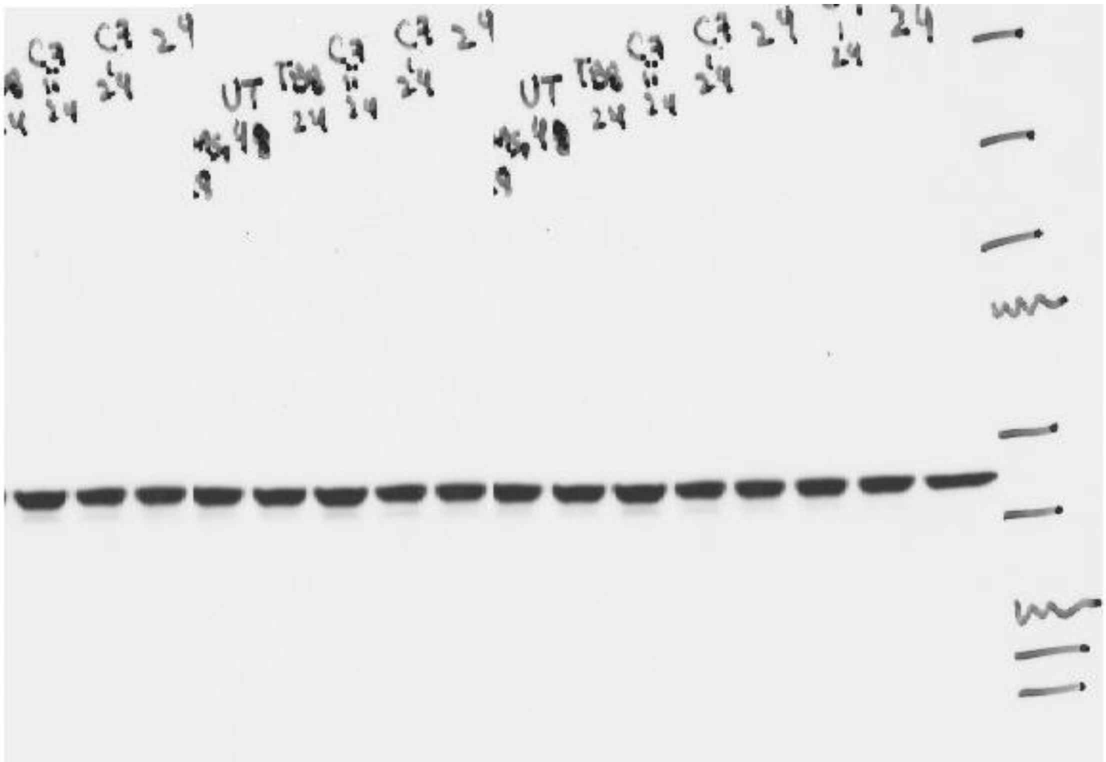

37kDa
